# The influence of home environment on 2-year-old Chinese children's language development: the mediating effect of executive function and the moderating effect of temperament

**DOI:** 10.3389/fpsyg.2024.1443419

**Published:** 2024-08-21

**Authors:** Siyao Qiu, Zhidan Wang

**Affiliations:** School of Education Science, Jiangsu Normal University, Xuzhou, China

**Keywords:** home environment, language ability, executive function, temperament, children

## Abstract

Prior research highlighted the effect of home environment on the language development of young children. Recent research has mainly discussed the moderating effect of personality traits like temperament. Nevertheless, the precise mechanism about the relationship between home environments to children's language development remains incompletely understood. This study explored how home environment impacts the language development of 2-year-old toddlers and the role of temperament and executive function in this relationship. We used the Chinese Child Adaptive Behavior Scale, the Temperament Scale for 1–3 years old of toddlers and the Home Environment Scale for Infants' and Toddlers' families to assess children's language development, temperament, and home environment. Simultaneously, the research used the Stroop-like day-night task and the multiple location search task to evaluate children's executive function. A total of 117 2-year-old children as well as their parents were involved in the study. The results revealed that home environment significantly predicts children's language ability with executive function as a mediating role. Temperament dimensions including extraversion, independence, reactivity, and social inhibition play a moderating role between home environment and executive function. The findings contributed to the improved implementation of home education tailored to children with different temperament traits, offering effective support for the cognitive and language development of young children.

## 1 Introduction

Home environment has a significant impact on the language development of young children (Peterson et al., 2019), which comprises both material and psychosocial dimensions (Sarsour et al., 2011). In addition, substantial research has presented the correlation between the executive function and language development of children at an early age (Shokrkon and Nicoladis, 2022). The early language development secures its foothold in executive function (Cartwright, 2012) and home environment (Bus et al., 1995). Nevertheless, developmental pathways among home environment, executive function, and language ability have been seldom explored (Segers et al., 2016). As a result, there lacks understanding toward the precise connections among these. Recent years have witnessed growing attention from researchers to the moderating effect of individual differences on young children (Cuevas et al., 2014; Wang et al., 2021), but no research has been made with a focus on the moderating effect of different temperaments and home environments on the individual executive function. Thus, it is worth exploring why different children benefit differently from their home environment in early childhood. This study sought to comprehensively explore the role of home environments in language development by examining whether executive function and temperament mediate or moderate the impact of environmental factors on language development in Chinese children aged 2 years.

### 1.1 Home environment and children's language development

Based on Brownian Brunner's (1979) ecosystem theory of developmental psychology, the microenvironment of human development is the institutions and groups with the most immediate, directly impact on children's development, such as family, school, religious institutions, neighborhood, and peers, among which home environment (HE) is the most influential microsystem on early childhood development.

Family socioeconomic status is a significant indicator of home environment (Chow et al., 2017). Family SES reflecting the resources and assets possessed by a household includes both material and intangible resources (Bradley and Corwyn, 2002). Prior studies have primarily examined the influence of the family socioeconomic status on early childhood language development (Bradley and Corwyn, 2002). Infants from high-SES families are exposed to more child-directed speech (Hart et al., 1997). However, those of low-SES families may be exposed to fewer, less diverse and simpler words and gain fewer conversational opportunities (Huttenlocher et al., 1991, 2010; Hoff, 2003; Rowe, 2012; Romeo et al., 2018). Similar conclusions have been drewn among Chinese families in which the impact of family SES has been verified in both Pinyin writing (Hoff, 2003; Dulay et al., 2018) and Chinese language (Zhang et al., 2013; Su et al., 2017). It is widely believed that the vocabulary disparity between those of low-SES households and high-SES households is resulted from different quantity and quality of their exposure to language (Ralph et al., 2020). In general, the home environments of those growing up in low-SES backgrounds are featured with chaotic organizations, the absence of structures and routines, various stressors (Hoff-Ginsberg, 1998; Hoff and Tian, 2005; Pace et al., 2017), and excessive background noises and crowding (Evans et al., 2005; Evans, 2006). This eventually makes the children to be inferior than their counterparts in access to language resources.

In sum, home environment provides young children with more opportunities and stimuli. Despite the empirical basis proving that home environment can predict the language development of young children, the exact mechanism for Chinese children aged 2 remains unclear and it has not been verified whether this conclusion can be equally applied to the children of different temperaments.

### 1.2 A potential mediator: executive function

Executive function (EF) means a group of top-down mental processes required for someone to concentrate when it is ill-advised, insufficient, or impossible to rely on instincts or intuition (Miller and Cohen, 2001). Three core EFs are universally recognized (Miyake et al., 2000; Lehto et al., 2003; Diamond, 2013): inhibition refers to the capacity to delay a well-learned prepotent response and replace it with a more appropriate one (Smith and Jonides, 1997; Baddeley, 1998); working memory represents the capability of keeping and controlling complex information in mind (Barkley, 2001); cognitive flexibility refers to the capacity to adapt individual behavior to the changing situation in a rapid, flexible manner (Davidson et al., 2006; Diamond, 2006).

Preschool years witness the boom of both Language and EF whose association has been verified by substantial (Shokrkon and Nicoladis, 2022). EF plays a big part in language skill acquisition and development of children as it prompts them to pay attention to different information streams, monitor errors, and make decisions accordingly (Diamond, 2013). EF, intrinsically involved in language functioning, is crucial in semantic control (Mirman and Britt, 2014). Although many cross-sectional (Gathercole and Pickering, 2000; Carlson et al., 2005; Kuhl, 2014) and longitudinal research (Gooch et al., 2016; Pérez-Pereira et al., 2020) as well as intervention studies (Guttentag et al., 2014; Jones et al., 2014) have demonstrated the bidirectional relation, few studies have concentrated on the direction of the developmental pathways between EF and language. Thus, their association remains unclear (Shokrkon and Nicoladis, 2022).

The relation between the home environment and executive function of children is well-documented (Sarsour et al., 2011). Home environment can exert both direct and indirect influences on a children's EF. For instance, a rich home literacy environment with regular book reading sessions may enhance the working memory and language processing capacity of a child (Boerma et al., 2017). Furthermore, positive parenting practices within the home environment, such as providing consistent routines and opportunities for problem-solving, can contribute to the inhibitory control of children (Sanders et al., 2019). In essence, home environment's direct influences on EF skills are evident through its capacity to shape the working memory, cognitive flexibility, and inhibitory control, and all of them are integral to language development (Diamond, 2013). With a growing number of findings about its significant roles, it becomes imperative to further examine the role of EF on the relationship between the HE and children language ability. Hence, this important variable is included in this study.

### 1.3 A potential moderator: temperament dimensions

As the biological differences of individuals, temperament is an enduring biological composition under the influence of heredity, maturation, and experiences (Rothbart et al., 2001; Rothbart and Derryberry, 2013). The evolution of children's executive function is influenced by both external and internal factors from the ecosystem (Bronfenbrenner, 1979; Tu and Yang, 2018). Regarding internal factors, temperament as a stable individual characteristic serves as the foundation for individual differences and can regulate the relation between the external environment and children's development (Rioux et al., 2016). Temperament serves as the innate foundation for children's reactivity and self-regulation (Bornstein et al., 2015). Children with certain temperament traits probably be more subject to environmental influences (Belsky et al., 2007). In studies on children aged 0–4, it has been found that children with high arousability temperament characteristics perform better in favorable environments compared to unfavorable environments. On the other hand, children with low arousability temperament characteristics show stable development in their executive functions and are less influenced by the environment (Willoughby et al., 2013). The temperament acts as a filter for stimuli, influencing the sensitivity of children to stimuli, and subsequently affecting their executive function (Xie et al., 2021). However, previous research has not provided answers to which components of temperament filter the impact of various environmental factors on executive function. The missing piece in executive function research is how temperament and environmental factors jointly influence the executive function of children (Suor et al., 2019). Hence, this potential moderator is included in this study.

### 1.4 Current study

To conclude, some studies have already discovered the significant impact of home environment on early linguistic competence development of young children. In addition, executive function of young children can predict their language development. However, the exact mechanism through which home environment influences the language development of children has not been fully understood, and there is limited evidence on the moderating effects of individual differences such as temperament dimensions. To close up the gap, it is predicted that executive function is a potential mediating factor, and children's temperament could function as a potential moderating factor. The study focused on 2-year-olds. Language development is pivotal for the future success of children (Visser-Bochane et al., 2020). Language acquisition at the age of 2 when children learn language and communication rapidly naturally serves as a crucial foundation for individual development (Suryanti et al., 2023). It has been well documented that the language development of children aged 2 to 3 varies with family factors (Linberg et al., 2020). In addition, given that 2-year-old children have not yet undergone formal schooling, the family serves as the primary setting for them to acquire fundamental skills and receive the necessary resources. As a result, home environment is the most influential microsystem on early childhood development (Leng et al., 2023). Therefore, selecting 2-year-old children as the subjects can deepen the understanding of early language development and optimize family education for young children with different types of temperament.

Specifically, the following hypotheses were developed and then tested: (1) EF acts as a mediator between the HE and 2-year-old children's language ability; (2) temperament plays the moderating role between HE and EF.

## 2 Methods

### 2.1 Participants

We selected 151 parents from a childcare institution of average local standards in Xuzhou City, Jiangsu Province, China, and permitted by the principals to account for potential dropouts and ensure robustness of our findings based on the sample sizes of previous related research (Wang et al., 2021; Xie et al., 2021). Both online and offline procedures were carried out to collect data. Online procedures were conducted through anonymous electronic questionnaires on *Wenjuanxing*, a public online platform. For the offline procedure, one-on-one tests were conducted with the children and their parents within the childcare institution. Online and offline sessions took place from September to October 2022.

Participants were all voluntary for the study, and written consent was submitted by parents. The study has been approved by the Ethical Committee of Jiangsu Normal University. 117 (77.5%) parents of children aged 2 (*M* = 31.06, *SD* = 3.27 months, 55 boys, 62 girls) responded effectively and contributed to the data analysis.

### 2.2 Procedures and measures

The measures of EF were organized into an offline test, and the measures of HE, temperament dimensions, and language ability were used to make an online questionnaire. The participants would receive a link of the questionaire and complete it at their convenience.

#### 2.2.1 Home environment

The three aspects of the home environment include the family socioeconomic status, family material environment, and family non-material environment. Specifically, each of these components was normalized and then summed to derive the overall *home environment* variable (Bradley and Corwyn, 2002).

*Family SES*. Parents were asked about their education level and vocational type (Linghao Xie and Fong, 2022). A total of seven education levels could be selected, from the lowest level of *not having attended school* (1 point) to the highest level of *master degree or higher* (7 points). A total of five types of occupations were classified while the lowest level was *temporary workers* (1 point) and the highest was *senior professionals* (5 points). The total score was obtained by adding the points gained by both mother and father from the above questions together. As a result, the total score should range from 4 to 24, and a higher total score demonstrates a higher family SES. Household income was not taken into account since it was proved that it could not accurately reect SES among the Chinese population by a prior report (Xu et al., 2006).

*Family material environment and family non-material environment*. These measures were derived from the Korean Home Environment Scale for Infants' and Toddlers' Homes (Kim et al., 2012) with Caldwell and Bradley's ECHOME scale (Caldwell and Bradley, 2001) as well as the research conducted by Kim and Gwak as its basis (Kim and Gwak, 2007). The scale used in this study has been adapted appropriately based on the local situation in China to assess the caregiving environment for children related to two factors: material environment (4 items) and non-material environment (4 items). The adaptations included modifications to reflect Chinese living habits, such as the types of media, videos, and toys commonly used. In these 8 items, the response scale of 6 items that were positively scored (e.g., *I usually read with my child a lot*) ranged from never true (1 point) to always true (5 points), 2 items that were negatively scores (e.g., *my child is usually exposed to media screen*) ranged from never (5 points) to everyday (1 point). The total score of each factor was used, while a high score indicates that those children are exposed to more diverse developmental environments than those of lower scores. The Cronbach's α of each factor ranged from 0.71 to 0.84.

#### 2.2.2 Children's language ability

We used a family self-report questionnaire to measure children's language ability, which was selected from the language development section in the Development of National Norms of Chinese Child Adaptive Behavior Scale (Yao and Gong, 1993).

After modification, our study encompasses several dimensions: First, it evaluates vocabulary size by assessing how many objects the child can name in daily life. Second, it examines self-identification by determining the child's awareness of their name and gender. Third, it assesses descriptive ability by evaluating the child's capability to describe pictures. Lastly, it considers the recognition of size and color by determining the child's ability to identify these attributes in objects. Finally, it evaluates functional vocabulary by assessing how many object uses the child can articulate. The Cronbach's α of each factor ranged from 0.90 to 0.99.

#### 2.2.3 Executive function

This study focuses on two primary components of executive function, that is, the working memory and inhibition. We used tasks that are suitable for children aged 2 and have been widely used and proved to have good measurement indicators.

- *Working memory*. This test is derived from multiple location search task (Carlson, 2016). The experimental materials consist of three cartoon stickers and six boxes with different shapes, colors, and sizes. The experimenter evenly placed six boxes with different colors and shapes on the table. The stickers were placed inside the boxes and covered with lids, and the entire process was performed while the children were watching. After closing the lids, the boxes were shuffled in random order, and then, the child was asked to find out the boxes that had the stickers hidden inside. The task consisted of 3 trials, with 1, 2, and 3 stickers at each level. Children will be awarded one point for correctly finding a sticker, with a total of six points available. The working memory of those of high scores is higher than those of lower scores.
- *Inhibition*. This test is derived from Stroop-like day-night task (Gerstadt et al., 1994). During the task, the experimenter first introduced two pictures to the participants, one representing day and the other representing night. In the case that the experimenter took out the day picture, the child needs to say “night”; when the night picture was shown, the child needs to say “day”. After ensuring that the child had understood the experiment rules, they would go through four practice trials (day-night-night-day). Once the child can answer correctly for all 4 consecutive trials, they will proceed to the formal experiment, which consists of 10 trials in total. Inhibition of those with high scores is higher than those with lower scores.

#### 2.2.4 Temperament dimensions

The parents were required to report the temperament of their children based on the 1- to 3-year-old Infants Temperament Scale (Li, 2011), which was developed based on the temperament characteristics of 1–3 year old in China. Seven temperament dimensions were explored in this study, namely, emotionality, activity, reactivity, social inhibition, focus, independence, and extroversion:

- Activity (e.g., *the child enjoys climbing and running around*), reactivity (e.g., *the child quickly engages in physical activities like crawling, walking, or running*), and social inhibition (e.g., *the child shows shyness when meeting new friends*) each consist of seven items rated on a scale from 1 (never true) to 5 (always true).
- Emotionality (e.g., *the child expresses strong emotions when their desires are not met immediately*) and focus (e.g., *the child can maintain focus on a task despite distractions such as doorbells or phone calls*) each include five items, also rated on a scale from 1 (never true) to 5 (always true).
- Independence (e.g., *the child comes up with unexpected ideas or expresses opinions different from adults*) and extroversion (e.g., *the child is comfortable meeting and playing with new children*) each comprise four items, which rated on a scale from 1 (never true) to 5 (always true).

A higher mean score on these items indicates a stronger manifestation of the specific temperament trait assessed. The Cronbach's α for each factor ranged from 0.74 to 0.84, demonstrating good internal consistency reliability.

## 3 Statistical analysis

First, descriptive statistics involving mean, standard deviations, and the range of all pivotal variables and bivariate relationships among Home Environment, Executive Function, and Language Ability were analyzed with SPSS version 26.0 on macOS 12.0 Monterey. Table 1 presents the descriptive statistics for children's age, home environment, language ability, and temperament, and Table 2 presents the inter-correlations between home environment, executive function, and language ability. Due to the skewed distribution of Executive Function, we have used Spearman's rank correlation coefficient to measure bivariate relations.

**Table 1:** Descriptive statistics for children's age, home environment, language ability, and temperaments.

**Variable**	***M***	***SD***	***Q*1**	***Median***	***Q*3**	***Range***
Age in months	31.06	3.27	29.00	31.00	34.00	24 − 36
Home environment	27.71	3.79	25.00	28.00	31.00	19 − 35
Language ability	18.87	3.78	17.00	20.00	22.00	6 − 22
Independence	14.64	2.59	13.00	15.00	16.00	3 − 20
Focus	3.31	0.52	3.00	3.40	3.60	2 − 4.6
Extroversion	14.10	2.73	12.00	14.00	16.00	4 − 20
Emotionality	2.58	0.70	2.00	2.60	3.00	0 − 4.6
Activity	3.66	0.66	3.14	3.57	4.00	2.29 − 5
Reactivity	3.90	0.65	3.43	3.86	4.43	2.29 − 5
Social inhibition	2.39	0.73	1.86	2.43	2.86	1 − 4.43
Executive function	8.07	5.31	5.00	9.00	14.00	0 − 16

**Table 2:** Inter-correlations between home environment, executive function, and language ability.

	**Home environment**	**Executive function**	**Language ability**
Home environment	1		
Executive function	0.279^*^	1	
	95%CI = [0.103, 0.438]		
Language ability	0.395^**^	0.331^**^	1
	95%CI = [0.230, 0.538]	95%CI = [0.160, 0.484]	

^**^p < 0.01, ^*^p < 0.5.

Second, to estimate both direct and indirect effects of Home Environment and Linguistic competence with Executive Function serving as the mediating variable, the Hayes PROCESS macro (Model 4) within the IBM SPSS Statistics 26.0 software package on macOS 12.0 Monterey was employed (Hayes, 2013). This analytical approach involved estimating these effects through the utilization of a bootstrapping technique with 5,000 resamples, facilitating the derivation of 95% confidence intervals for the coefficients associated with each path. The validity of mediation was determined by examining whether the value ‘zero fell outside the confines of the 95% confidence interval.

**Figure 1:**
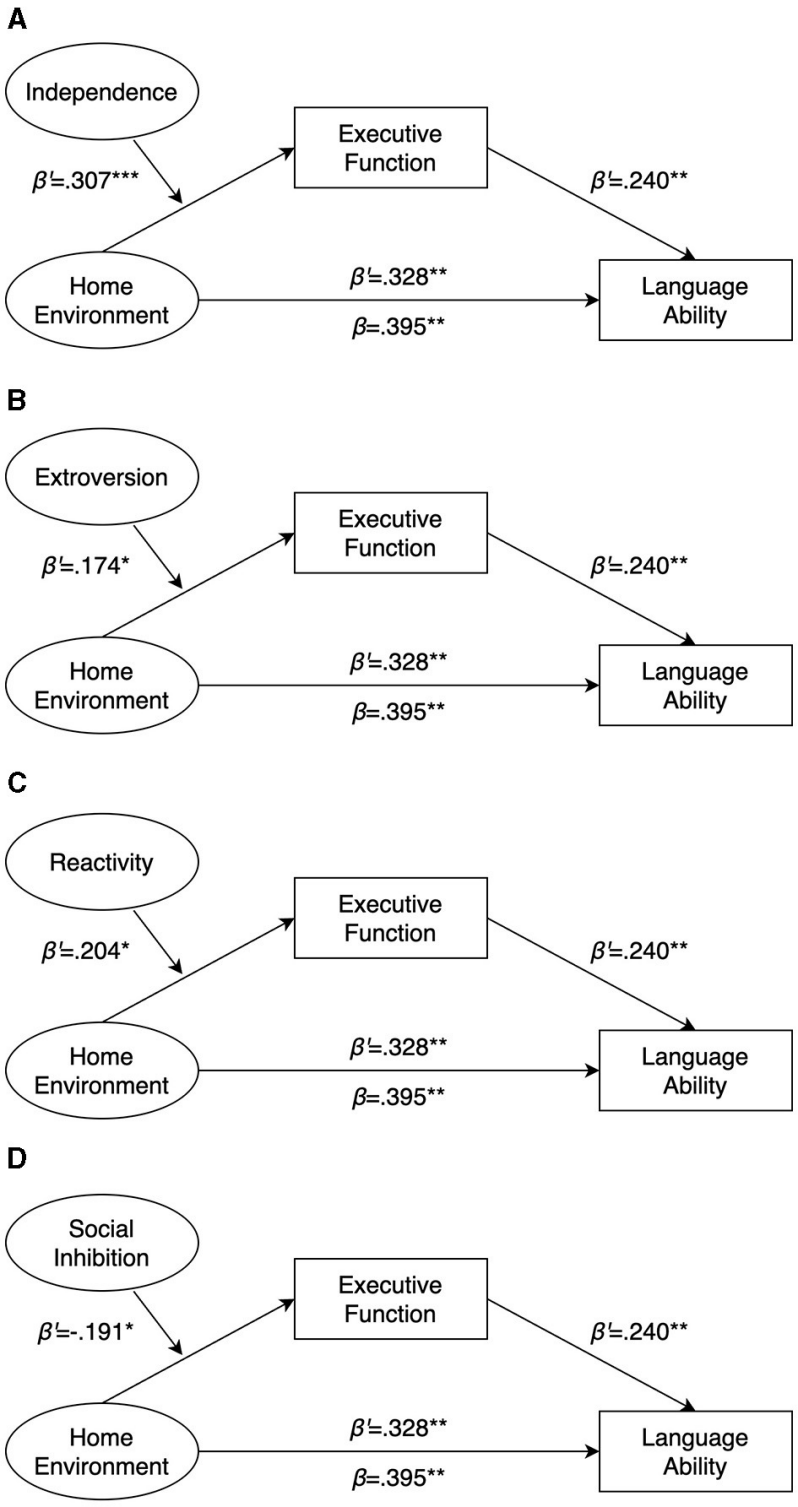
Mediated moderation model of executive function on language ability through home environment with temperament variables as moderators. **(A)** Independence as temperament variable. **(B)** Independence as temperament variable. **(C)** Reactivity as temperament variable. **(D)** Social inhibition as temperament variable. ^*^p < 0.1, ^**^p < 0.05, ^***^p < 0.01.

Then, to investigate the moderating effect of temperament variables on the relationship between Home Environment and Executive Function, the Hayes PROCESS macro (Model 7) was applied. This analysis involved the assessment of the effects of Home Environment, temperament variables, and their interactive effects. Results are presented in Figure 1. Furthermore, 95% Confidence Intervals were computed for these interaction effects. Similar to the mediation analysis described earlier, the validity of moderation was determined by examining whether the value “zero” fell beyond the boundaries of the 95% Confidence Interval for the interactions (Hayes, 2013).

**Figure 2:**
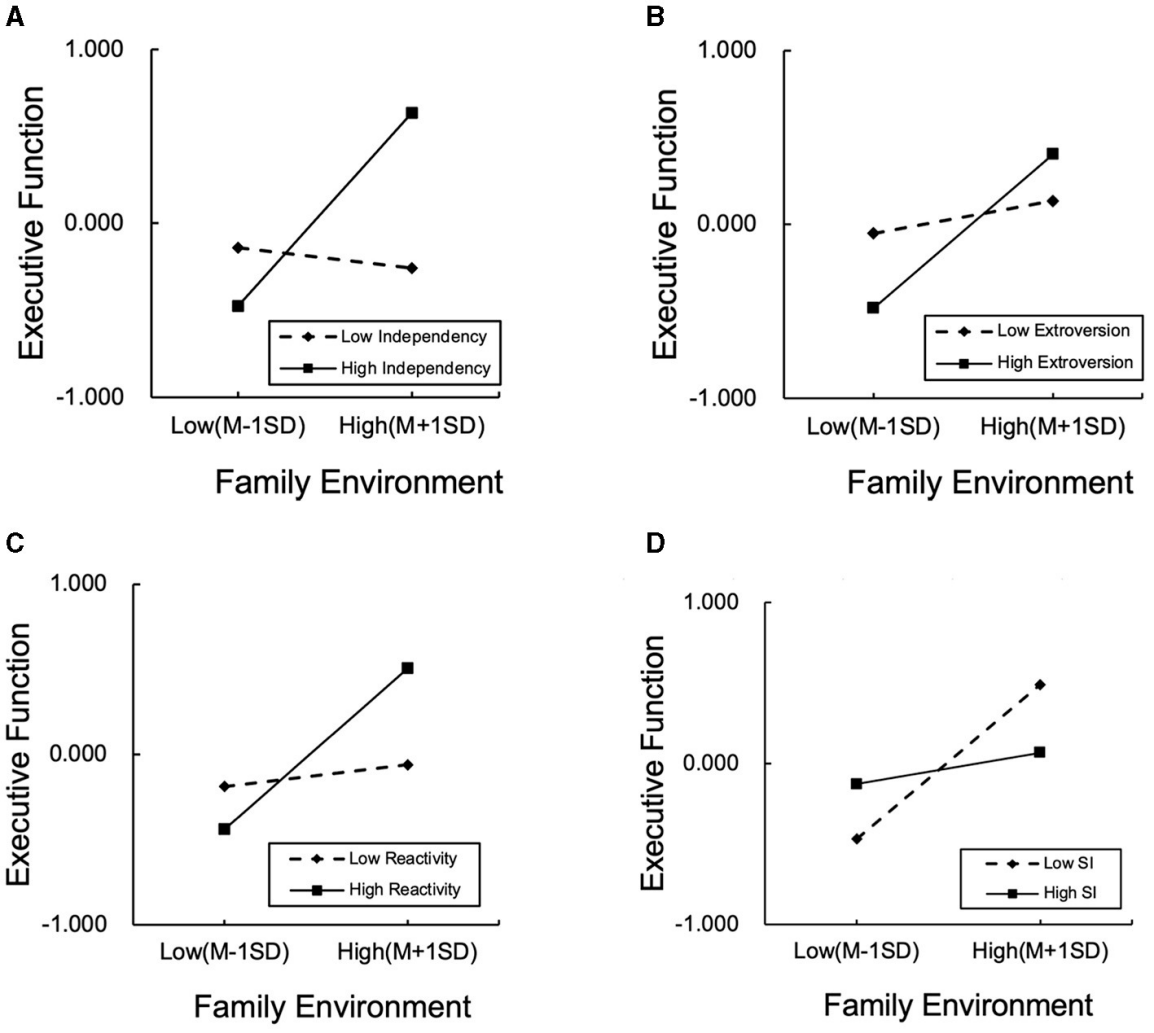
Home environment by executive function, with temperaments (*independence, extroversion, reactivity, and social Inhibition*) as moderators. **(A)** Independence. **(B)** Extroversion. **(C)** Reactivity. **(D)** Social Inhibition.

Finally, the simple slop test was used to test and facilitate the presentation of the complex-mediated moderation analyses. Four linear prediction graphs (see Figure 2) were constituted by two separate graphs depicting Executive Function (*y*-axis) as a function of Home Environment at different temperament levels.

## 4 Results

### 4.1 Descriptive statistics and correlations

The descriptive analyses and Pearson correlation results are shown in Tables 1, 2. Home environment was significantly and positively associated with both executive function (*r*(117) = 0.279, *p* < 0.05) and language ability (*r*(117) = 0.395, *p* < 0.01). In addition, executive function was also positively related to language ability (*r*(117) = 0.331, *p* < 0.01).

### 4.2 Mediating role of executive function

We use SPSS macro-PROCESS (Hayes, 2013) (model 4) to explore the mediating effect of executive function on the link between home environment and language ability (Table 3). Home environment was significantly positively correlated with language ability with a regression coefficient of β = 0.39, *p* < 0.01. When the mediating variable was considered, executive function was significantly positively correlated to home environment (β = 0.28, *p* < 0.01), and language ability was also correlated to executive function (β = 0.240, *p* < 0.01) and was significantly positively correlated with home environment (β = 0.33, *p* < 0.01).

**Table 3:** Results for the mediating effect of executive function (HE, home environment; LA, language ability; EF, executive function).

**Outcome variable**		***R***	***R* ^2^**	***F***	**df(1)**	***p***	**β**	***t***
Language ability	HE	0.3949	0.1560	21.2534	1	<0.0001	0.39	4.61^**^
Executive function	HE	0.2789	0.0778	9.7007	1	0.0023	0.28	3.11^**^
Language ability	HE	0.4573	0.2091	15.0690	2	<0.0001	0.33	3.78^**^
	EF						0.24	2.77^**^

^**^p < 0.01.

The upper and lower bounds of the bootstrap (95% confidence interval) for the mediating effect of executive function did not contain zero (Table 4), demonstrating that executive function plays a mediating role in the link between home environment and language ability, where mediating effect accounted for 17% of the total effect. It is important to note that this mediation effect is partial, indicating that in addition to executive function, other factors may also play a role in the relationship between home environment and language development. Future research could explore these additional factors to fully understand the complex relationship.

**Table 4:** Mediation effect breakdown.

**Home environment → Language ability**	**β**	**Boot SE**	**Boot LLCI**	**Boot ULCI**	**Percentage**
Total effect	0.3949	0.0857	0.2253	0.5646	-
Direct effect	0.3280	0.0867	0.1562	0.4998	83.06%
Indirect effect (executive function)	0.0669	0.0362	0.0124	0.1529	16.94%

### 4.3 Moderation effect of temperaments

Using SPSS macro-PROCESS, the moderation effect of seven temperament traits was estimated (Table 5) within the mediation model. The direct correlation between home environment and language ability was consistent with the mediation model discussed earlier. Five of the seven temperament traits (independence, extroversion, reactivity and social inhibition) acted as moderators in the regression equation, and three did not have significant moderating effect: the interaction between focus and home environment was not significant (β = 0.041, *p*>0.05), as was the interaction between activity (β = 0.135, *p*>0.05) and emotionality (β = −0.017, *p*>0.05). These findings indicates that with moderators of independence, extroversion, reactivity and social inhibition, executive function could still mediate the relationship between home environment and language ability.

**Table 5:** Testing the moderating effect of temperament variables on home environment.

**Temp Var**	**HE β**	**Temp. β**	**Interaction β**	**Lower**	**Upper**	***R* ^2^**	***F***
Indep.	0.248^**^	0.140	0.307^***^	0.130	0.485	0.172	7.810^***^
Extro.	0.268^**^	−0.040	0.174^*^	0.009	0.339	0.113	4.785^**^
React.	0.268^**^	0.078	0.204^*^	0.013	0.395	0.120	5.125^**^
Social.	0.288^**^	−0.021	−0.191^*^	−0.347	−0.035	0.124	5.328^**^
Focus.	0.258^**^	0.134	0.041	−0.144	0.227	0.098	4.111^**^
Act.	0.285^**^	−0.051	0.135	−0.038	0.309	0.099	4.142^**^
Emo.	0.277^**^	−0.159	−0.017	−0.183	0.150	0.103	4.310^**^

^***^p < 0.001, ^**^p < 0.01, ^*^p < 0.05.

Moreover, the interaction of home environment and independence (β = 0.307, *p* < 0.001), extroversion (β = 0.174, *p* < 0.05) and reactivity (β = 0.204, *p* < 0.05) had significantly positive effect on executive function, while the interaction of home environment and social inhibition (β = −0.191, *p* < 0.05) had significantly negative effect. To further explore the moderating role of temperament, the Johnson-Neyman method (Hayes, 2013) was adopted for a simple slope analysis (see Table 6).

**Table 6:** Marginal effects on linear predictions of executive function.

	**Independence**		**Extroversion**		**Reactivity**		**Social inhibition**	
	**Slope**	***p***	**Slope**	***p***	**Slope**	***p***	**Slope**	***p***
M - 1SD	−0.059	0.644	0.094	0.457	0.064	0.623	0.479^***^	<0.001
Mean	0.248^**^	0.005	0.268^**^	0.003	0.268^**^	0.004	0.289^**^	0.002
M + 1SD	0.557^***^	<0.001	0.442^***^	<0.001	0.472^***^	<0.001	0.097	0.405

^***^p < 0.001, ^**^p < 0.01, ^*^p < 0.05.

Figures 2A–C show that this effect was significant for children with higher levels of independence (simple slope = 0.557, *p* < 0.001), extroversion (simple slope = 0.442, *p* < 0.001), and reactivity (simple slope = 0.472, *p* < 0.001). For children with low independence, extroversion, and reactivity, this effect was not significant. Figure 2D shows a significant effect for children with lower social inhibition (simple slope = 0.479, *p* < 0.001). Thus, high levels of independence, extroversion, and reactivity, together with low level of social inhibition, strengthen the association between family environment and execution function.

## 5 Discussion

Prior studies have explored the relationship between home environment and linguistic competence (Chow et al., 2017; Shokrkon and Nicoladis, 2022), home environment and executive function (Sarsour et al., 2011; Han et al., 2023), and executive function and language ability (Segers et al., 2016; Shokrkon and Nicoladis, 2022). However, there is a notable gap in the research studies regarding the simultaneous examination of these three variables. The current study is aimed to close up this gap by investigating the mediating effects of executive function and the moderating role of seven dimensions of temperament. The results of this study deepen the understanding toward the influence of the above-mentioned factors on the language development of children, which contributes to gain a deeper appreciation of the intricate dynamics involved in early childhood language development and provide customized supports for different children.

First, our findings align with Bronfenbrenner's ecological systems theory, which emphasizes that development is influenced by various environmental systems. We found that executive function partially mediates the relationship between the home environment and language development, supporting our first hypothesis. According to Bronfenbrenner's model, the home environment, part of the microsystem, provides essential resources and interactions that promote children's executive function and language development. Higher quality home environments are better equipped to offer consistent routines, positive parenting practices, and material resources, which are crucial for enriching activities that stimulate both executive function and language development (Kroenke, 2008). This supports the notion that the immediate environment plays a pivotal role in child development. Furthermore, executive function serves as an intermediary mechanism that partially mediates the impact of the home environment on language ability by increasing children's engagement in relevant interactions and activities (Bohlmann and Downer, 2016). This partial mediation suggests that other factors may also play a role, aligning with the ecological model's acknowledgment of multiple interacting influences on development. Future research could explore these additional factors to fully understand the complex relationship.

To illustrate, children with better inhibition capacity would behave more appropriately during their conversation with adults, which helps them retain adult vocabulary and syntax (Hanno and Surrain, 2019). Moreover, children more flexible in cognition would be more skilled in the application of the variable linguistic rules (Gathercole and Baddeley, 1989). To illustrate, the same words may have different meanings in different contexts, while some linguistic conventions can only be used in certain contexts (Hanno and Surrain, 2019). Moreover, evidence reveals that working memory contributes to children‘s vocabulary development, especially its phonological short-term component (Gathercole and Baddeley, 1989; Gathercole and Pickering, 2000; Gathercole, 2006).

Secondly, we found that out of the seven types of temperament, four types (i.e., independence, extroversion, reactivity, and social inhibition) were moderating variables to moderate the impact of home environment on executive function. Our hypothesis (2) has been supported partially. Some research has indicated that children with extroverted traits and children with negative emotional and active traits are more susceptible to environmental influences (Pluess and Belsky, 2013). Individuals with high extraversion are able to actively seek problem-solving strategies from their environment, but they are also easily attracted to novelty and more sensitive to rewards, which can lead to impulsivity and a lack of persistence in uninteresting tasks (Xie et al., 2021). This is consistent with the findings of our study on extraversion but inconsistent with the results on emotionality and activity. This may be due to the emotionality and activity dimensions indirectly affect executive functions by other factors or mechanisms. In addition, emotionality may have some other effects on the relationship between home environment and executive functions, rather than having a direct moderating effect. Such effects may require more complex research methods to uncover.

Extraversion at different levels may be influential in guiding cognition (Campbell et al., 2011). It is believed that executive functions are improved by biological processes in respect to extraversion (Rammsayer, 1998). Regarding independence dimension, children with high level of independence tend to have a greater ability to self-regulate and adapt to independent learning. They are more likely to utilize external support within the family, which contributes to an improvement in their executive function (Wang and Zhou, 2021). In terms of reactivity, children with high reactivity are more likely to exhibit a heightened sensitivity and positive response to external stimuli (Ellis et al., 2011). This means that they are more easily attracted to various learning opportunities, challenges, and new experiences in their environment, as they are more keenly aware of these changes, thereby enhancing their cognitive development. With regard to social inhibition, children with low social inhibition may indicate more positive social engagement, allowing them to effectively utilize social resources in their family environment, leading to improved executive function. Lastly, concerning the dimension of focus, it is possible that there is some overlap between it and executive function, so its moderating effect is not significant, which needs to further explore.

In conclusion, our study found that home environment significantly predicts children's language ability, with executive function playing a mediating role and temperament moderating the impact of home environment on executive function. This suggests that a rich family environment can promote the linguistic competence development of children aged 2. Children who are more independent, extraverted, responsive, and socially uninhibited are more likely to actively engage in language interactions using family resources. This participation influences 2-year-old children's language development by enhancing their executive function. The findings of this study can contribute to better family education for children with different temperaments and provide personalized and effective support for their cognitive and language development.

Notably, several limitations existed in our study. First, our study focused exclusively on Chinese children, which may limit the generalizability of the findings to other cultural contexts and populations. Second, as all of our participants came from an urban city, their SES gap may not be typical across the country. Hence, families from a wider range of communities or countries should be included in future studies. Third, language and temperament measurements relied on self-reported data from parents, which may result in response bias and social desirability bias. Further research can adopt objective measures or observational data, which could enhance the validity of the results. Finally, the influence of children's temperament may be a mixture rather than a single factor. This study primarily focuses on examining the moderating role of a single temperament trait. Future research can consider investigating the combined effects of multiple temperament traits.

## 6 Conclusion

Our study demonstrates a significant positive association between home environment and language development in 2-year-olds, with executive function serving as a key mediator. The influence of the home environment on language ability is further moderated by temperamental traits such as independence, extroversion, reactivity, and social inhibition. These insights suggest that enhancing social support and nurturing specific personality traits could mitigate the adverse effects of a less-than-ideal home environment, thereby promoting better language outcomes. The research underscores the need for further investigation into how individual differences can be leveraged to optimize early childhood development, potentially leading to more effective interventions and support strategies.

## Data Availability

The raw data supporting the conclusions of this article will be made available by the authors, without undue reservation.
